# Coexistence of Small Duct Type and Biliary Adenofibroma–Associated Large Duct Type Intrahepatic Cholangiocarcinoma within a Single Lesion: A Case Report

**DOI:** 10.70352/scrj.cr.26-0269

**Published:** 2026-08-25

**Authors:** Sachiko Ishikami, Sunao Uemura, Teppei Tokumaru, Shuta Tamura, Motoyasu Tabuchi, Rika Yoshimatsu, Manabu Matsumoto, Jun Iwata, Takehiro Okabayashi

**Affiliations:** 1 Department of Gastroenterological Surgery, Kochi Health Sciences Center, Kochi, Kochi, Japan; 2 Department of Radiology, Kochi Health Sciences Center, Kochi, Kochi, Japan; 3 Department of Diagnostic Pathology, Kochi Health Sciences Center, Kochi, Kochi, Japan

**Keywords:** biliary adenofibroma, intrahepatic cholangiocarcinoma, small duct type, large duct type, cholangiolocarcinoma, malignant transformation

## Abstract

**INTRODUCTION:**

Biliary adenofibroma (BAF) is a rare biliary epithelial tumor with malignant potential, and it has been reported in association with intrahepatic cholangiocarcinoma (iCCA). Although the malignant transformation of BAF has been described in sporadic case reports, the coexistence of distinct histopathological subtypes of iCCA in association with a single BAF lesion is exceedingly rare.

**CASE PRESENTATION:**

An asymptomatic 75-year-old man was incidentally found to have a 35-mm hepatic mass in hepatic segment 7 during an evaluation for prostate cancer. Imaging studies suggested iCCA, and partial hepatectomy was performed. Histopathological examination revealed 2 distinct tumor components: a small duct type iCCA with cholangiolocellular subtype features and a large duct type iCCA arising from BAF. A distinct transition zone was identified between the BAF and large duct type iCCA components, whereas the small duct type iCCA component was present adjacent to the BAF-associated large duct type component. The postoperative course was uneventful, and the patient remained disease-free 10 months after surgery.

**CONCLUSIONS:**

We report a rare case of the coexistence of small duct type iCCA and BAF-associated large duct type iCCA within a single lesion. This case highlights the biological heterogeneity and malignant potential of BAF-associated biliary neoplasia and underscores the importance of complete resection and careful follow-up.

## INTRODUCTION

Biliary adenofibroma (BAF) is an extremely rare epithelial tumor of the liver that resembles von Meyenburg complexes.^1)^ Although BAF was initially regarded as a hamartomatous lesion, increasing evidence suggests that BAF possesses malignant potential, as supported by accumulated clinicopathological evidence^2–5)^ and recent systematic reviews.^6–8)^

Intrahepatic cholangiocarcinoma (iCCA) is classified into 2 major histopathological subtypes—large duct type and small duct type—which differ in their clinicopathological and molecular characteristics.^9–11)^ Large duct type iCCA typically arises from established precursor lesions such as biliary intraepithelial neoplasia or intraductal papillary neoplasm of the bile duct,^12–14)^ whereas well-defined precursor lesions for small duct type iCCA remain uncertain.^10,11,15)^

Most previously reported cases of iCCA arising from BAF have not been systematically classified according to the current small duct type and large duct type framework.^6)^ Therefore, the relationship between BAF and the histopathological subtypes of iCCA remains incompletely understood. Herein, we report an exceptionally rare case of the coexistence of small duct type and BAF-associated large duct type iCCA within a single hepatic lesion, each accompanied by a distinct histological transition zone.

## CASE PRESENTATION

An asymptomatic 75-year-old man was incidentally found to have a hepatic mass during an evaluation for prostate cancer at another hospital and was referred to our institution for further management. His medical history included internal carotid artery stenting for carotid artery stenosis, endoscopic treatment for gastric cancer, and chronic hepatitis B virus infection. Hormonal therapy for prostate cancer was initiated 2 months prior, and the prostate-specific antigen level decreased to within the normal range. Other tumor markers, including carcinoembryonic antigen, carbohydrate antigen 19-9, alpha-fetoprotein, and protein induced by vitamin K absence-II, were within normal limits. Biochemical tests, including hepatobiliary enzyme levels, showed no abnormalities; liver function was well preserved, with Child–Pugh class A (score 5).

**Fig. 1:**
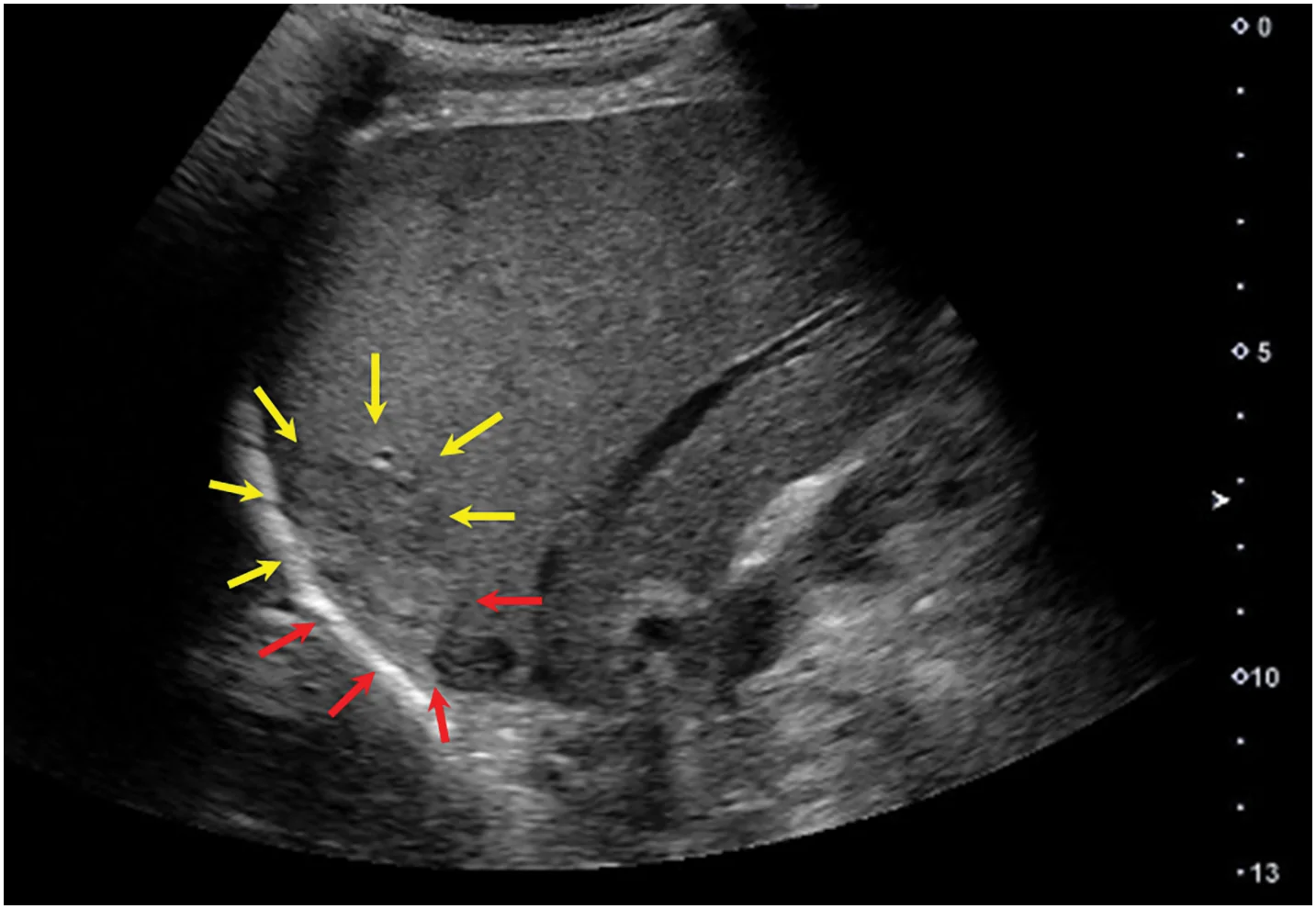
Preoperative AUS. AUS reveals a well-demarcated, dumbbell-shaped mass (maximum diameter, 35 mm) in the posterior segment of the liver. The inner portion appears slightly hyperechoic (red arrows), whereas the outer portion shows isoechoic to hypoechoic features (yellow arrows). AUS, abdominal ultrasonography

**Fig. 2:**
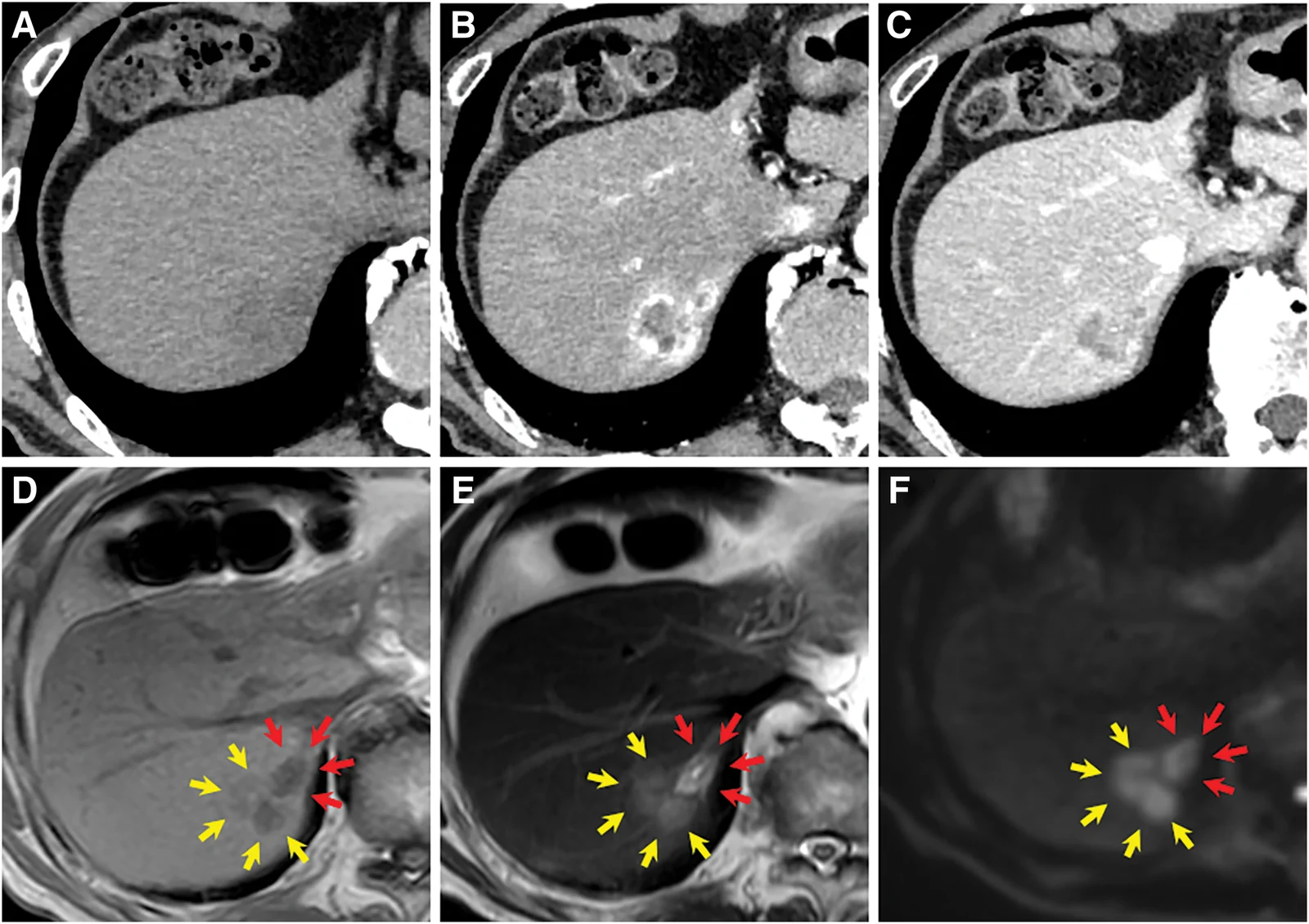
Preoperative radiological findings. (**A**) Abdominal CT reveals a low-attenuation tumor measuring approximately 30 mm in diameter. (**B**) Contrast-enhanced abdominal CT shows peripheral enhancement in the early phase, and (**C**) persistent internal enhancement in the delayed phase. (**D**) MRI shows slight hypointensity on T1-weighted images; compared with the inner portion (red arrows), which histopathologically corresponded to the BAF-associated large duct type iCCA component, the outer portion (yellow arrows), corresponding to the small duct type iCCA component, appears relatively isointense. (**E**) On T2-weighted images, the inner portion (red arrows) shows high signal intensity, whereas the outer portion (yellow arrows) exhibits iso- to slightly high signal intensity. (**F**) On diffusion-weighted imaging, both the inner (red arrows) and outer portions (yellow arrows) demonstrate mildly increased signal intensity, although the lesion exhibits a lobulated morphology. BAF, biliary adenofibroma; iCCA, intrahepatic cholangiocarcinoma

**Fig. 3:**
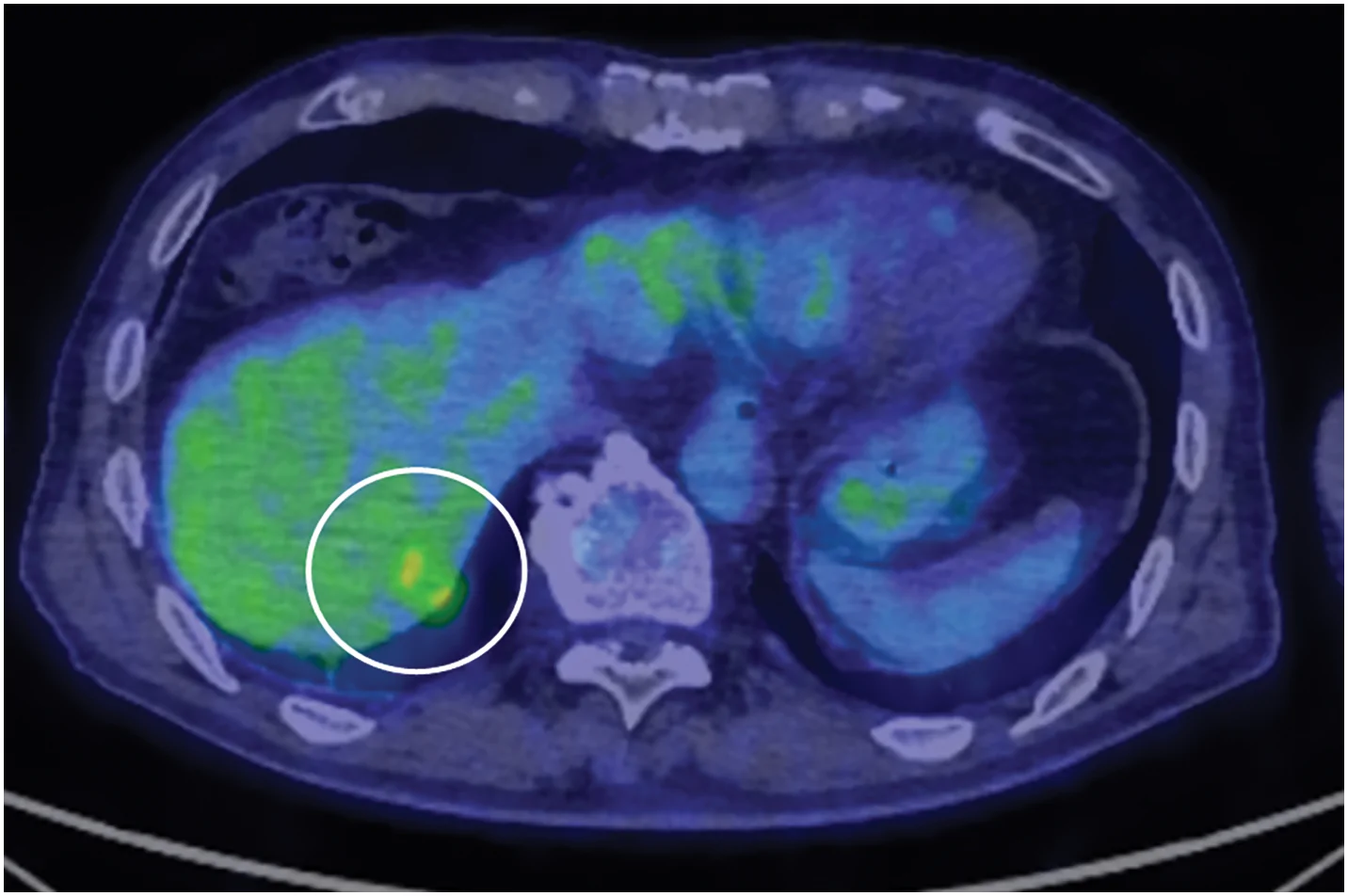
Preoperative ^18^F-FDG PET-CT. ^18^F-FDG PET-CT reveals FDG uptake in the hepatic tumor (maximum standardized uptake value, 4.3), and the tumor is outlined in white. FDG, fluorodeoxyglucose

Abdominal ultrasonography (AUS) revealed a well-demarcated bilobed (dumbbell-shaped) mass measuring 35 mm in maximum diameter within the hepatic segment 7. The lesion exhibited heterogeneous internal echogenicity, with a hyperechoic inner component and a relatively hypoechoic outer component, suggesting distinct tissue compositions (**Fig. 1**). Contrast-enhanced CT revealed peripheral enhancement in the early phase, persistent internal enhancement in the delayed phase, and Glissonian pedicles coursing through the tumor (**Fig. 2A**–**2C**). No lymph node metastases were observed. MRI revealed slight hypointensity on T1-weighted images, slight hyperintensity on T2-weighted images, and mildly increased signal intensity on diffusion-weighted images (**Fig. 2D**–**2F**). On ^18^F-fluorodeoxyglucose PET-CT, abnormal fluorodeoxyglucose uptake was observed in the hepatic mass, with a maximum standardized uptake value of 4.3, whereas no abnormal uptake was detected in other organs, including the prostate or lymph nodes (**Fig. 3**). A metastatic liver tumor was considered unlikely, given that liver metastasis from prostate cancer is exceptionally rare, and no primary lesions suggestive of metastasis from other organs were identified. Although AUS and MRI raised the possibility that the tumor comprised 2 distinct components, contrast-enhanced CT revealed continuous enhancement throughout the entire lesion from the periphery, with the Glissonian pedicles coursing through the interior of the tumor, strongly suggesting a primary hepatic origin. Therefore, the tumor was preoperatively diagnosed as iCCA. Although the patient was receiving hormonal therapy for prostate cancer, curative treatment could be deferred for >1 year under continued hormonal therapy; therefore, surgical resection of the hepatic tumor was planned.

**Fig. 4:**
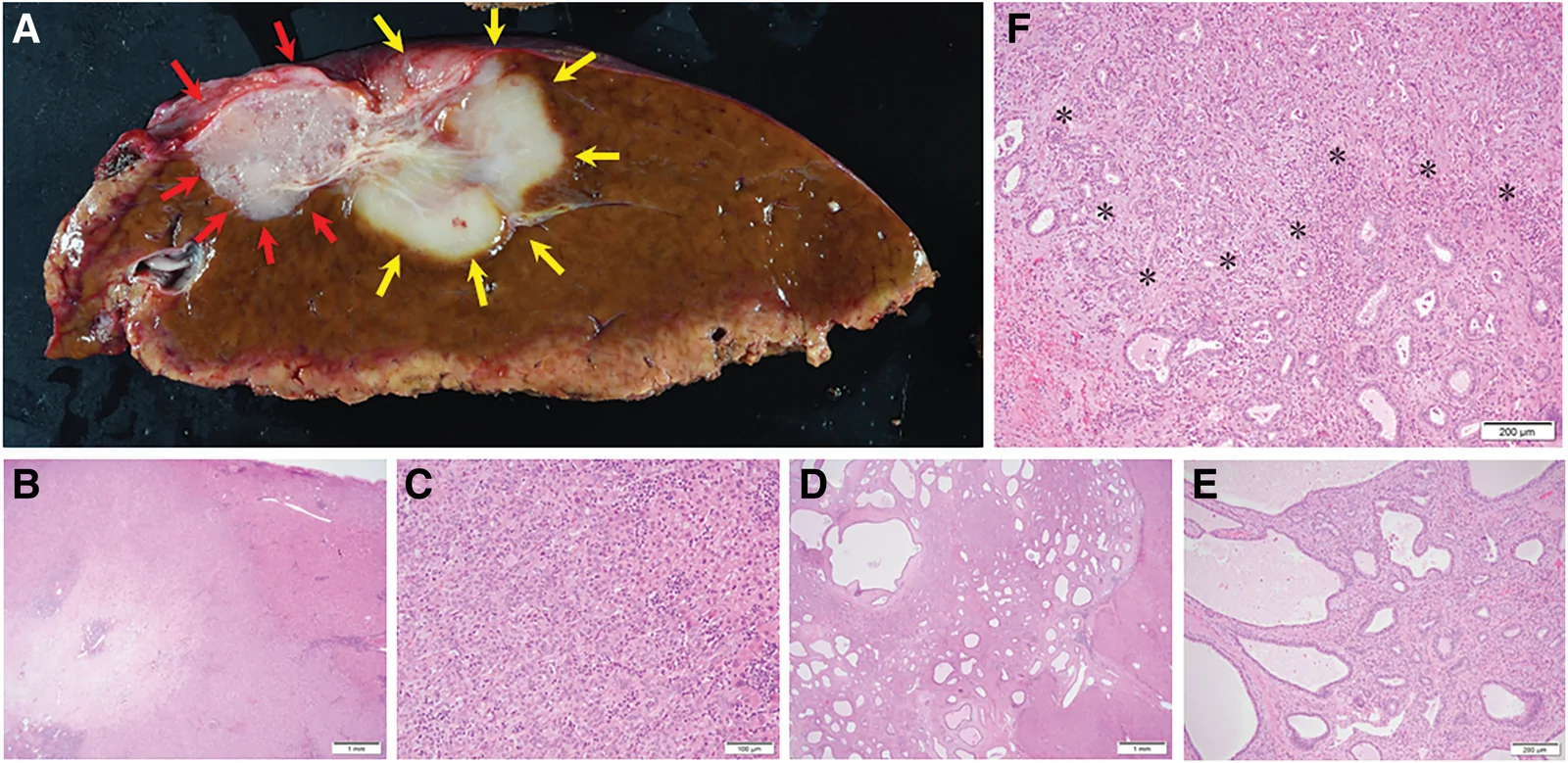
Resected specimen and histopathological findings of the hepatic tumor. (**A**) Macroscopic examination of the resected specimen shows a yellowish-white solid tumor (yellow arrows) and a reddish, spongiform tumor (red arrows) measuring 29 × 18 × 24 mm. Orientation: the right side of the image represents the patient’s left side. (**B**, **C**) Hematoxylin and eosin staining of the yellowish-white solid tumor component shows poorly formed glandular structures composed of tumor cells with relatively abundant cytoplasm. Scale bars, 1 mm (**B**) and 100 μm (**C**). (**D**, **E**) The reddish, spongiform tumor component consists of a tubular tumor with well-defined glandular formations arising from cuboidal to columnar epithelium, accompanied by abundant and relatively uniform fibrous stroma. Scale bars, 1 mm (**D**) and 200 μm (**E**). (**F**) A distinct transitional zone is identified between the BAF and the large duct type iCCA components in the area below the asterisks, while the small duct type iCCA component in the area above the asterisks shows close spatial association with the BAF-associated lesion. Scale bar, 200 μm. BAF, biliary adenofibroma; iCCA, intrahepatic cholangiocarcinoma

The patient underwent partial hepatectomy of the hepatic segment 7. Regional lymph node dissection was not performed owing to the absence of radiologically suspicious lymphadenopathy. The operative time was 152 min, with an intraoperative blood loss of 290 mL, and no blood transfusion was required. On gross examination of the resected specimen, the tumor measured 29 × 18 × 24 mm. Two distinct tumor components were identified: a yellowish-white solid tumor and a reddish, spongiform tumor (**Fig. 4A**). Histopathologically, the former component consisted of poorly formed glandular structures composed of tumor cells with relatively abundant cytoplasm (**Fig. 4B** and **4C**). Immunohistochemical staining revealed positivity for cytokeratin (CK) 7 and CK19 and negativity for hepatocyte paraffin 1 and arginase-1, confirming the biliary epithelial phenotype. Scattered cluster of differentiation 56-positive cells were also observed. Expression of epithelial membrane antigens was detected in a membranous-to-cytoplasmic manner. Based on these findings, the component was diagnosed as a small duct type iCCA with cholangiolocellular subtype features. The latter component consisted of a tubular tumor with well-defined glandular formations arising from the cuboidal to columnar epithelium, accompanied by abundant and relatively uniform fibrous stroma (**Fig. 4D** and **4E**). Focal mucin production was also observed. Additional histochemical and immunohistochemical analyses revealed focal Alcian blue positivity within the stromal mucinous areas and focal MUC6 expression in the epithelial cells, whereas MUC5AC and S100P were negative. Only a few scattered S100-positive stromal cells were identified in the stroma. Taken together with the characteristic morphology and continuity with the BAF component, these findings supported the diagnosis of BAF with associated large duct type iCCA arising from BAF. Distinct transitional zones were identified in both the small duct type iCCA and the BAF-associated large duct type iCCA components (**Fig. 4F**). No vascular invasion was identified, and the closest surgical margin was 12 mm. The background liver exhibited mild portal lymphocytic inflammation without interface activity and mild lobular inflammation, with centrilobular macrovesicular-predominant steatosis (10%–20%) and suspected hepatocellular ballooning. Focal bridging fibrosis was present, but no perivenular or pericellular fibrosis was identified. Overall, these findings were suggestive of steatohepatitis.

The postoperative course was uneventful, and the patient was discharged on POD 8 without complications. For iCCA, the patient was carefully followed up without adjuvant chemotherapy, with contrast-enhanced CT imaging and tumor marker assessments performed every 3 months. Definitive radiotherapy for prostate cancer was administered 7 months after surgery. The patient remained alive without iCCA recurrence at 10 months postoperatively.

## DISCUSSION

This case provides rare pathological evidence of the coexistence of small duct type iCCA and BAF-associated large duct type iCCA within a single lesion. Increasing evidence suggests that BAF has clinically meaningful malignant potential.

Tsui et al. first reported BAF in 1993 as a benign lesion characterized by tubulocystic and tubuloglandular bile duct–like structures embedded in fibrous stroma, while noting morphological features suggestive of a neoplastic process and the possibility of malignant epithelial transformation if left untreated.^1)^ Subsequent clinicopathological studies and case reports have repeatedly described epithelial atypia, dysplasia, and overt carcinoma arising within or contiguous with BAF, thereby challenging its traditional classification as a purely benign biliary lesion.^2–6)^ Histologically, several reports have emphasized the coexistence of benign and malignant components, sometimes with recognizable transition zones, supporting malignant transformation rather than simple collision.^2,4,6)^ Molecular data remain limited; however, Thompson et al. reported a *CDKN2A* loss-of-function mutation in malignant BAF, suggesting a possible role of cell cycle dysregulation in progression.^2)^ In addition, Sturm et al. identified shared *TP53* and *KIT* polymorphisms in benign and malignant components, which may support clonal relatedness but should not be interpreted as established driver events.^4)^ Importantly, a systematic review of 55 reported BAF cases found invasive carcinoma in 52.7% (29/55) of patients; most patients with available follow-up data were disease-free after resection, and only a minority experienced relapse or disease-related death, indicating clinically meaningful malignant potential with heterogeneous outcomes.^6)^ Taken together, the current evidence supports the view that BAF is a true biliary neoplasm with genuine malignant potential rather than a simple hamartomatous process.

iCCA is currently classified into 2 major histopathological subtypes—large duct type and small duct type—which differ in their histological features, molecular characteristics, etiology, and presumed cells of origin.^9–11)^ Large duct type iCCA typically arises through a multistep carcinogenic process from well-established precursor lesions such as biliary intraepithelial neoplasia or intraductal papillary neoplasm of the bile duct.^12–14)^ In contrast, well-defined precursor lesions for small duct type iCCA remain controversial, and this subtype is generally considered to arise *de novo* from peripheral bile ducts or hepatic progenitor cells.^10,11,15)^ Recent studies have shown that these 2 subtypes exhibit distinct immune microenvironments and patterns of intratumoral heterogeneity.^16,17)^

In this case, histopathological examination demonstrated the coexistence of small duct type iCCA and BAF-associated large duct type iCCA within a single lesion, each showing a distinct transition zone. We conducted a comprehensive literature search of PubMed, Embase, and Web of Science using the search terms “biliary adenofibroma” AND (“cholangiocarcinoma” OR “intrahepatic cholangiocarcinoma” OR “malignant transformation”) for the period from January 1993 to January 2025. Although a systematic review by Mattiolo et al. identified multiple cases of malignant transformation arising from BAF, none demonstrated the synchronous development of both small and large duct type iCCA from a single lesion.^6)^ To the best of our knowledge, this is the first documented case demonstrating the coexistence of small duct type iCCA and BAF-associated large duct type iCCA within a single lesion. Importantly, the currently available literature does not allow a definitive conclusion regarding the predominant histological subtype of BAF-associated carcinoma. The present case highlights the biological heterogeneity of BAF-associated biliary neoplasia.

The complex histological architecture observed in this case provides an important context for recently proposed conceptual frameworks of BAF-associated biliary neoplasia. Masetto et al. proposed that tubulocystic carcinoma of the bile ducts is a distinct histological entity arising from adenofibroma-type lesions, emphasizing a characteristic tubular and cystic architecture accompanied by relatively uniform fibrous stroma.^18)^ In addition, Zen and Luchini suggested that BAF should be interpreted not as a purely benign lesion but as part of a broader adenofibroma–tubulocystic carcinoma–cholangiocarcinoma continuum reflecting progressive neoplastic transformation.^19)^ In this context, this case is notable in that the coexistence of a small duct type iCCA component broadens the histopathological spectrum of BAF-associated biliary neoplasia beyond that currently encompassed by tubulocystic carcinoma of the bile duct. Of note, the BAF-associated large duct type iCCA component in our case was negative for MUC5AC and S100P, both of which are commonly expressed in conventional large duct type iCCA.^10,11,15)^ Despite this atypical immunoprofile, the diagnosis was supported by the characteristic morphology and continuity with the BAF component. These findings raise the possibility that BAF-associated large duct type iCCA may show an immunophenotype distinct from that of conventional large duct type iCCA. Further accumulation of well-characterized cases will be required to better define its diagnostic spectrum.

From a clinical perspective, the preoperative diagnosis of BAF-associated carcinoma remains challenging. No specific radiological features can reliably distinguish BAF or its malignant transformation from conventional iCCA in routine imaging studies.^8,20,21)^ In this case, although AUS and MRI suggested the presence of 2 distinct components, contrast-enhanced CT revealed enhancement throughout the lesion from the periphery, and definitive preoperative discrimination between the 2 histological subtypes was not possible. Furthermore, fluorodeoxyglucose PET-CT may show only mild-to-moderate uptake in BAF-associated malignancies, as observed in our case (maximum standardized uptake value, 4.3).^20,21)^ Therefore, such lesions should be managed as suspected iCCA in clinical practice, and a definitive diagnosis relies on careful histopathological evaluation of the resected specimen, with adequate sampling of different morphological areas.

Complete surgical resection with negative margins remains the only potentially curative treatment for BAF with malignant transformation^7)^ and early-stage iCCA.^22)^ In this case, partial hepatectomy of hepatic segment 7 was performed, and the patient’s postoperative course was uneventful. The patient remained recurrence-free for 10 months after surgery. Because the optimal surveillance strategy for BAF-associated iCCA remains unknown, long-term follow-up with periodic contrast-enhanced CT or MRI and tumor marker monitoring should be considered, particularly in view of the potential for delayed recurrence.^6)^ Although adjuvant chemotherapy was not administered because complete resection was achieved and the pathological features were favorable, the patient’s history of hormonal therapy and radiotherapy for prostate cancer suggests that adjuvant chemotherapy may be warranted in cases with adverse prognostic factors such as positive surgical margins, lymph node metastasis, or poorly differentiated histology.

## CONCLUSIONS

We report a rare case demonstrating the coexistence of small duct type iCCA and BAF-associated large duct type iCCA within a single hepatic lesion, each accompanied by a distinct histological transition zone. This case highlights the biological heterogeneity and malignant potential of BAF-associated biliary neoplasia and broadens its histopathological spectrum beyond currently recognized classifications. Complete surgical resection with negative margins and careful long-term follow-up are essential for the appropriate management of such lesions.

## DECLARATIONS

### Availability of data and materials

All data generated or analyzed during this study are included in the published article.
